# Enhanced lignin degradation by *Irpex lacteus* through expanded sterilization further improved the fermentation quality and microbial community during the silage preservation process

**DOI:** 10.1186/s40643-024-00730-2

**Published:** 2024-01-22

**Authors:** Xiaohui Cao, Rui Cai, Sasa Zuo, Dongze Niu, Fuyu Yang, Chuncheng Xu

**Affiliations:** 1 College of Engineering, China Agricultural University, (East Campus), 17 Qing-Hua-Dong-Lu, Haidian District, Beijing, 100083 People’s Republic of China; 2 School of Environmental Science and Engineering, Huazhong University of Science and Technology, Wuhan, 430074 Hubei Province China; 3 Changzhou Key Laboratory of Biomass Green, Safe and High Value Utilization Technology, Institute of Urban and Rural Mining, Changzhou University, Changzhou, 213164 China; 4 College of Grassland Science and Technology, China Agricultural University, Beijing, 100093 People’s Republic of China

**Keywords:** Expansion, *Irpex lacteus*, Lignin, Ensiling, Microbial community, In vitro production

## Abstract

Traditional autoclaving, slow degradation rate and preservation of biomass treated by fungi are the main factors restricting biological treatment. In our previous studies, strains with high efficiency and selective lignin degradation ability were obtained. To further solve the limiting factors of biological treatment, this paper proposed a composite treatment technology, which could replace autoclaves for fungal treatment and improve the preservation and utilization of fungal-pretreated straw. The autoclaved and expanded buckwheat straw were, respectively, degraded by *Irpex lacteus* for 14 days (CIL, EIL), followed by ensiling of raw materials (CK) and biodegraded straw of CIL and EIL samples with *Lactobacillus plantarum* for different days, respectively (CP, CIP, EIP). An expansion led to lactic acid bacteria, mold, and yeast of the samples below the detection line, and aerobic bacteria was significantly reduced, indicating a positive sterilization effect. Expansion before *I. lacteus* significantly enhanced lignin selective degradation by about 6%, and the absolute content of natural detergent solute was about 5% higher than that of the CIL. Moreover, EIL decreased pH by producing higher organic acids. The combination treatment created favorable conditions for ensiling. During ensiling, EIP silage produced high lactic acid about 26.83 g/kg DM and the highest acetic acid about 22.35 g/kg DM, and the pH value could be stable at 4.50. Expansion before *I. lacteus* optimized the microbial community for ensiling, resulting in EIP silage co-dominated by *Lactobacillus*, *Pediococcus* and *Weissella*, whereas only *Lactobacillus* was always dominant in CP and CIP silage. *Clavispora* gradually replaced *Irpex* in EIP silage, which potentially promoted lactic acid bacteria growth and acetic acid production. In vitro gas production (IVGP) in EIL was increased by 30% relative to CK and was higher than 24% in CIL. The role of expansion was more significant after ensiling, the IVGP in EIP was increased by 22% relative to CP, while that in CIP silage was only increased by 9%. Silage of fungal-treated samples reduced methane emissions by 28% to 31%. The study demonstrated that expansion provides advantages for fungal colonization and delignification, and further improves the microbial community and fermentation quality for silage, enhancing the nutrition and utilization value. This has practical application value for scaling up biological treatment and preserving the fungal-treated lignocellulose.

**Graphical Abstract:**

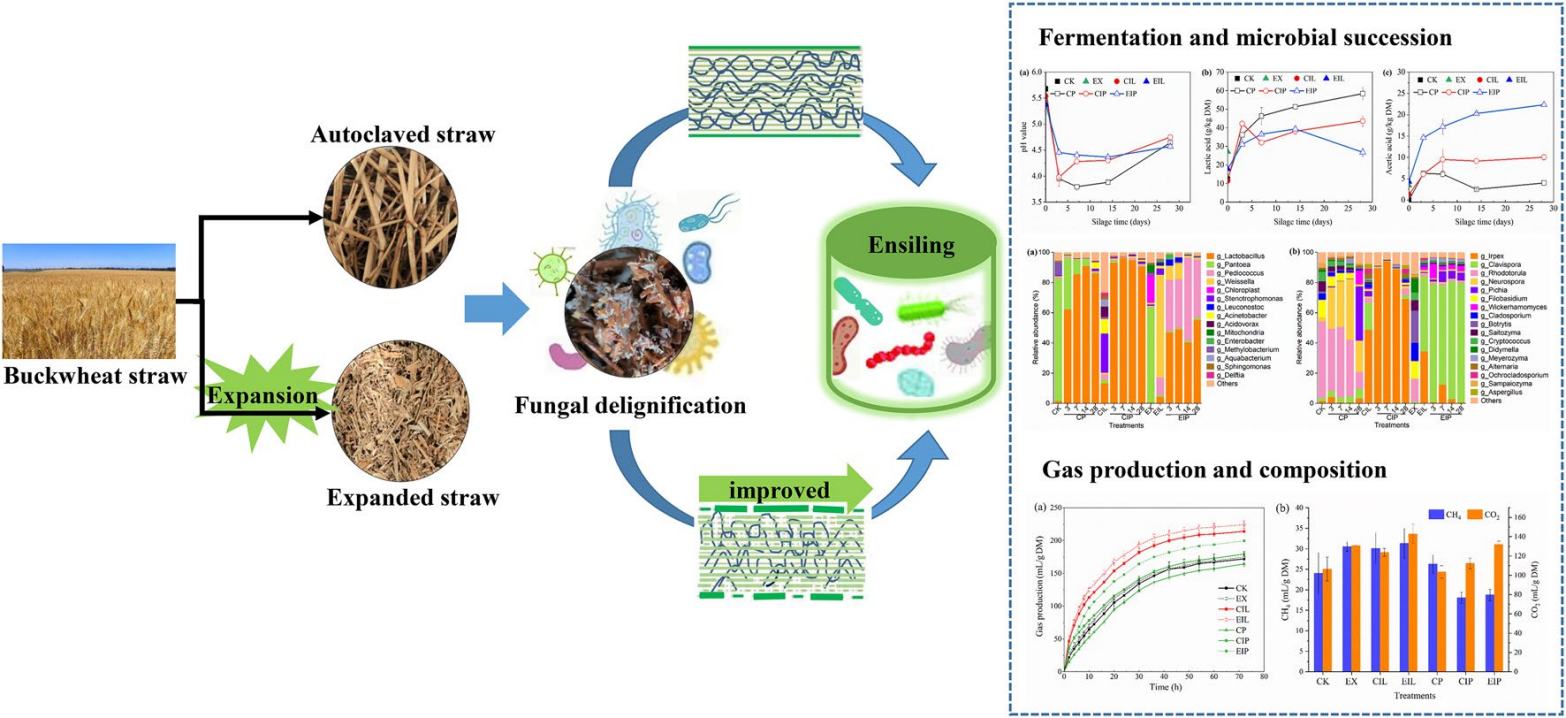

**Supplementary Information:**

The online version contains supplementary material available at 10.1186/s40643-024-00730-2.

## Introduction

The development and utilization of renewable biomass resources are an important way to cope with energy demand and food competition between humans and animals, such as straw biomass production of ethanol and ruminant feed (Chen et al. 2022; Kumar et al. 2020), and microalgae production of biohydrogen (Sallam et al. 2022). For China, with a wide area of crop cultivation, the rational utilization of agricultural by-products such as straw is the best choice to protect the environment, develop sustainable agriculture and maintain economic growth. This is because straw is a high molecular weight biopolymer rich in natural polysaccharides such as cellulose, hemicellulose, and lignin, which are linked by glycosidic bonds as support for plant cells (El-Mossalamy et al. 2021). However, the degree of lignification of straw is generally high, and lignin wraps around hemicellulose and cellulose to form a dense vascular bundle resistance structure (Woiciechowski et al. 2020). Owing to the difficulty of lignin degradation under natural conditions, direct feeding or fuel conversion may lead to low digestibility and incomplete energy conversion, resulting in a decrease in added value. Our previous research has shown that white-rot fungi can effectively improve the feed efficiency of straw resources by reducing lignin content, breaking down cell wall resistance and improving the conversion rate of straw (Niu et al. 2020; Zheng et al. 2022; Zuo et al. 2018). However, the production of saturated steam during autoclaving is not only energy-intensive, but also constitutes high capital costs for autoclaves. These problems are crucial reasons why fungal treatment is unattractive to industry. Developing a method that combines with fungal treatment, if it can replace sterilization while destroying cell wall resistance, would be beneficial for promoting the development of biological treatment technology.

Expansion is a kind of thermo-mechanical pretreatment, which involves high friction, extrusion, and shear between the feeding screw, straw and the inner wall of the machine to provide additional heat and energy, forming a high temperature and pressure environment (Guiao et al. 2022; Souza et al. 2022). After the pressure is released at discharge port, the straw explosion occurs, causing depolymerization of macromolecules and enhancing their biodegradability (Cao et al. 2024; Duque et al. 2017). This technology has low energy consumption, is a continuous process, and is easy to scale-up, which has been applied in multiple industries (Debiagi et al. 2020; Duque et al. 2017). Our recent findings revealed that expansion offered an advantageous platform for fungal treatment and promoted white-rot fungi preferentially contact lignin by achieving depolymerization and separation of cellulose fibers, partial hydrolysis of hemicellulose, and redistribution of lignin. We conclude that autoclaving expanded straw led to faster mycelial growth of white-rot fungi and a higher lignin degradation (Cao et al. 2023). However, autoclaving increased the complexity and energy consumption, therefore, we considered whether the severe conditions generated by the expansion process could be regarded as a potential sterilization step for fungal inoculation. It is necessary to know the survival microbial community on the expanded straw and reveal the impact of co-cultivated with more aggressive *I. lacteus* on the degradation of lignocellulose and the fermentation system. However, although white-rot fungi degradation can improve nutritional value, its palatability and preservation are poor. Owing to the discontinuity of agricultural production and the continuity of industrialization, it is essential to provide an economic storage mode for continuous supply of fungal-treated biomass. In addition, the termination of fungal activity after successful lignin degradation is also critical to retaining the nutritional value of biomass (Mao et al. 2018).

Ensiling is a cost-effective and eco-friendly strategy for large-scale and long-term preservation of wet biomass (Tao et al. 2020). The combination of anaerobic conditions and acidity protects the biomass from the proliferation of deleterious bacteria and fungi (Yang et al. 2001), which can enhance the storage duration of biomass with minimum nutrient losses and improve palatability as feed. Therefore, ensiling not only ensures a continuous supply for ruminants but also maintains bioenergy supply chains. However, due to the complex microbial sources after fungal degradation, whether lactic acid bacteria could quickly dominate and whether expanded straw has more advantages for silage remains unclear. Next-generation sequencing can reveal the microbial community and related functions and provide insights into fermentation regulation.

It is hypothesized that expansion can be regarded as a sterilization process, further, the disruption of lignocellulose structure and exposure of lignin caused by expansion would enhance the competitiveness of *I. lacteus* growth and achieve rapid colonization. Subsequently, the enhancement of cell wall degradation and changes in the microbial community caused by the coupling of expansion and *I. lacteus* may improve silage quality and preservation of fungal-treated biomass. Thus, the objective of this study was to determine the effect of expansion sterilization for delignification and microbial community during fermentation by *I. lacteus* and investigate the effect of delignification on the ensiling process by analyzing fermentation quality and microbial community.

## Materials and methods

### Materials and microorganism preparation

The harvested buckwheat straw, provided by Sichuan Province, China, was air-dried and stored in a cool and dry place. *I. lacteus* (China General Microbiological Culture Collection Center, CGMCC-5.809) was deposited in our laboratory, and was preserved on potato dextrose agar (PDA) (Nissui, Tokyo, Japan) slants at 4 ℃. The strain was cultured on PDA plates for 7 days at 28 °C. Ten discs (1 cm in diameter) of the plate culture were filled in a 250 mL Erlenmeyer flask with 200 mL of potato dextrose medium and cultivated at 28 ℃ and 150 rpm for 7 days as liquid species. *Lactobacillus plantarum* (GenBank No. SUB3928584) used in ensiling was obtained from our laboratory, which was taken out of the cryostat tube and inoculated in de Man Rogosa Sharp agar (MRS) (Difco Laboratories, Detroit, MI, USA) liquid medium for activation for two generations.

### Expansion and fungal pretreatment of buckwheat straw

The expansion process of producing expanded straw (EX) is consistent with the description of our previous study (Cao et al. 2023). The expanded sample was immediately transferred to an ultra-clean workbench, and the moisture content was approximately 48% by a rapid moisture tester. Each bag should contain 120 g of samples and 40 g of sterile water and then inoculated with 60 mL of homogeneous mycelium of fungi, obtaining about 71% moisture content (labeled EIL). The raw buckwheat straw (CK) was cut to 1–2 cm and the moisture content was also adjusted to 61%. After fully kneading by hand at room temperature, stored at 4 °C for 24 h to let the water completely penetrate. Then 160 g of fresh substrate in each bag was autoclaved at 121 ℃. After cooling to room temperature, inoculating 60 mL of homogeneous mycelium of fungi to the sterilized straw, obtaining about 71% moisture content (labeled CIL). All fungal treatments were fermented at 28 °C for 14 days. Three bags of CIL and EIL samples were dried at 65 °C for 48 h and then weighed, determining the dry matter (DM) loss and chemical composition. The remaining samples were silage experiments.

### Ensiling

Fungal-treated samples in CIL and EIL were packed into vacuum-packed bag silo (25 width × 36 cm height), with 1 × 10^6^ colony-forming units of *Lactobacillus plantarum* g^−1^ wet substrate (labeled CIP and EIP, respectively). Raw buckwheat straw silage was used as a control (labeled CP). Three bags of each treatment were collected at 3, 7, 14, and 28 days of storage for fermentation characteristic analysis.

### Chemical analysis

The first subsample of all treatments was oven-dried at 65 °C for 48 h. The contents of neutral detergent fiber (NDF), acid detergent fiber (ADF), acid detergent lignin (ADL), hemicellulose (HC), and cellulose (CL) were determined as described by Zuo et al. (2018). The water-soluble carbohydrate (WSC) content was determined according to Zhao et al. (2021). Ash by incineration at 550 ℃ for 3 h. Absolute numbers were calculated on the remaining air-dry matter after fungal incubation corrected for DM content in dried material, and DM loss during silage is not accounted for.

### Fermentation profile and microbial populations

The second subsample (10 g) was mixed with 90 mL of deionized water and extracted at 4 °C for 4 h to prepare water extracts, and then filtered. The filtrates were used to determine pH (S20K, Mettler Toledo, Greifensee, Switzerland) and organic acids (lactic, acetic, propionic, and butyric acids) by liquid chromatography system (column, Hitachi GL C-610H; oven temperature 50 ℃; mobile phase, 3 mmol/l HClO_4_, 1.0 ml/min; detector, L-7420 SUV-Vis).

The filtered silage extract was serially diluted from 10^–2^ to 10^–7^ with distilled water and spread onto plates for the counts of molds, yeasts, and aerobic bacteria in the samples (Niu et al. 2020). The count of lactic acid bacteria (LAB) was determined on MRS incubated anaerobically for 48 h at 37 °C. Molds and yeasts were counted on PDA after incubation at 28 °C for 48 h and aerobic bacteria were estimated on nutrient agar (NA) (Nissui-Seiyaku Ltd., Tokyo, Japan) at 30 °C for 48 h under aerobic conditions. Microbial numbers were expressed as colony-forming unit (cfu) per gram on fresh matter (FM) basis.

### Bacterial and fungal community

The total DNA of all samples was extracted using E.Z.N.A. Soil DNA Kit (Omega Bio-tek, Inc., USA) following the protocols outlined by the manufacturer. The V3-V4 hypervariable regions of bacterial 16S rDNA genes were amplified with the universal primers 338 F (ACTCCTACGGGAGGCAGCAG) and 806 R (GGACTACNNGGGTATCTAAT) (Cai et al. 2023a). For fungi, the ITS1 region was targeted using universal primers ITS1-F (CTTGGTCATTTAGAGGAAGTAA) and ITS2 (TGCGTTCTTCATCGATGC). The amplicon pools were paired-end sequenced through the Illumina Miseq PE300 platform (Illumine Inc., San Deigo, CA) and analyzed by Beijing Allwegene Technology Co. Ltd (Beijing, China) after qualification. The raw sequences were merged using PEAR (v0.9.6), followed by removing the redundancy using VSEARCH (v2.13.3) (Liu et al. 2021). The operational taxonomic units (OTUs) at 97% sequence similarity level were clustered using UPARSE. Subsequently, the obtained sequences were assigned to amplicon sequence variants (ASVs). The ASVs of the bacteria and fungi were annotated using SILVA (release 138) and Unite (release 8.2) databases, respectively. Functional gene prediction based on Kyoto Encyclopedia of Genes and Genomes (KEGG) databases was conducted using PICRUSt2 (Cai et al. 2023b; Douglas et al. 2020).

### In vitro gas production (IVGP_72_) technique

In vitro fermentation was performed according to Menke et al. (1979). Rumen fluid of fistulated non-lactating cows fed a grass silage-based diet was mixed with a buffer solution under anaerobic conditions. Air-dried samples (about 220 mg) were incubated in a 100 mL glass syringe for 72 h at 39 ◦C, with 30 mL of the buffered rumen fluid. The IVGP_72_ was recorded at 0, 2, 4, 6, 8, 10, 12, 16, 20, 24, 30, 36, 42, 48, 60, and 72 h of incubation and released total gas was used to measure the methane (CH_4_) and carbon dioxide (CO_2_) concentrations by gas chromatography. All cultures had three replicates.

### Statistical analysis

The data for microbial numbers were transformed by log_10_ and presented on a fresh matter (FM) basis. The statistical analysis was analyzed by one-way ANOVA followed by Tukey’s multiple range tests and declared when *P* < 0.05 using IBM SPSS 25 (IBM SPSS Inc., Chicago, IL, USA).

## Results

### Analysis of chemical composition

The chemical composition of buckwheat straw before and after expansion and fungal degradation is shown in Table 1. Lignin and HC contents in EX were significantly lower than that in CK (*P* < 0.05), and NDS and WSC content in EX increased significantly (*P* < 0.05). After inoculation with *I. lacteus* fermentation, the DM loss in EIL was about 13.6%, significantly higher than 10.2% in CIL (*P* < 0.05). The degradation rate of lignin in EIL was 6% higher than in CIL, and NDS content was higher in EIL (*P* < 0.05).

**Table 1:** Chemical composition of *I. lacteus* treated different buckwheat straw for 14 days

Item Treatments	Relative content (g/kg DM)				SEM	*p*-value	Absolute content (g)		SEM	*p*-value
	CK	EX	CIL	EIL			CIL	EIL		
DM loss (%)	–	–	–	–			10.2^b^	13.6^a^	0.85	0.015
NDS	319.8^ab^	349.2^a^	299.9^b^	324.1^ab^	6.59	0.034	268.4^b^	280.4^a^	3.17	0.034
Lignin	80.2^b^	73.7^c^	86.6^a^	77.9^c^	1.80	< 0.001	77.6^a^	67.4^b^	2.39	0.004
CL	462.1	459.0	473.6	476.6	3.77	0.338	423.8	411.7	3.32	0.170
HC	137.8^ab^	118.2^b^	139.4^a^	121.4^b^	3.70	0.015	122.0^b^	105.0^a^	4.21	0.020
WSC	116.3^a^	123.0^a^	67.5^b^	47.9^c^	9.73	< 0.001	60.5^a^	41.4^b^	4.59	0.007
Ash	51.1^c^	61.9^b^	63.0^b^	76.2^a^	2.73	< 0.001	56.4^b^	65.9^a^	2.27	0.005

CK, raw buckwheat straw; EX, expanded straw; CIL and EIL represent CK and EX groups treated with *I. lacteus* for 14 days respectively. NDS, natural detergent solute; CL, cellulose; HC, hemicellulose; WSC, water-soluble carbohydrate. SEM, standard error of means

Table 2 (at the end of the document) displays the chemical composition of feedstock and both biodegraded samples for different days of ensiling, and the absolute content indicates the effect caused by the removal of fungal degradation. A small change in fiber composition was observed in CP and EIP silage, while NDS, HC, and CL contents were observed loss in CIP silage, especially on day 28. EIP silage had significantly the highest NDS and WSC contents, and the lowest lignin and HC contents than others during ensiling (*P* < 0.05). The WSC content of all silages significantly decreased with the increase of silage days (*P* < 0.05).

**Table 2:** Chemical composition of different buckwheat straw ensiled with *L. plantarum*

Unit		Relative content (g/kg DM)				Absolute content (g)			
Silage days (d)		3	7	14	28	3	7	14	28
Item	Treatments								
NDS	CP	315.2^ABa^	313.0^Aa^	305.6^Aab^	299.9^b^	315.2^Aa^	313.0^Aa^	305.6^Aab^	299.9^Ab^
	CIP	302.8^Bab^	290.2^Bb^	292.8^Bab^	310.9^a^	271.8^Bab^	259.7^Cb^	262.0^Cb^	278.1^Ba^
	EIP	323.0^Ab^	314.6^Aab^	314.7^Aab^	312.0^a^	279.4^B^	272.1^AB^	272.2^AB^	270.0^B^
SEM		1.79				3.35			
*P-value*	*A*	< 0.001				< 0.001			
	*D*	0.016				0.093			
	*A* × *D*	0.002				0.028			
ADL	CP	80.7^ABb^	81.2^ABb^	84.2^Cab^	86.4^Ba^	80.7^Ab^	81.2^Ab^	84.2^Ab^	86.4^Aa^
	CIP	83.8^Ab^	84.1^Ab^	94.4^Aa^	97.5^Aa^	75.0^Bb^	75.2^Bb^	84.5^Aa^	87.2^Aa^
	EIP	79.1^Bab^	77.7^Bb^	77.5^Bb^	82.5^Ba^	68.4^Cab^	67.2^Cb^	67.0^Bb^	71.2^Ba^
SEM		1.03				1.26			
*P-value*	*A*	< 0.001				< 0.001			
	*D*	< 0.001				< 0.001			
	*A* × *D*	< 0.001				< 0.001			
CL	CP	464.2^b^	465.6^Bb^	469.5^b^	481.5^Aa^	464.2^Ab^	465.6^Ab^	469.5^Ab^	481.5^Aa^
	CIP	468.4^ab^	484.1^Aa^	472.1^ab^	457.3^Bb^	419.3^Bab^	433.2^Ba^	422.5^Bab^	409.3^Bb^
	EIP	464.8^b^	473.0^ABa^	473.5^a^	471.7^Aab^	402.1^Cb^	408.8^Ca^	409.6^Ca^	408.1^Bab^
SEM		1.38				4.75			
*P-value*	*A*	0.962				< 0.001			
	*D*	0.013				0.019			
	*A* × *D*	< 0.001				< 0.001			
HC	CP	142.7^a^	140.2^a^	140.0^a^	132.1^b^	142.7^Aa^	140.2^ABa^	140.0^Aa^	132.1^Ab^
	CIP	144.9^a^	141.7^a^	140.6^a^	133.2^b^	129.7^Ba^	126.8^Bab^	125.9^Bb^	119.3^Bc^
	EIP	133.0	134.6	134.3	133.7	104.9^C^	115.0^B^	116.3^B^	115.7^B^
SEM		1.05				1.81			
*P-value*	*A*	0.030				< 0.001			
	*D*	0.059				0.032			
	*A* × *D*	0.594				0.486			
WSC	CP	91.9^Aa^	80. 7^Aab^	71.4^Abc^	58.7^Ac^	91.9^Aa^	80. 7^Aab^	71.4^Abc^	58.7^Ac^
	CIP	16.0^Ba^	13.0^Bc^	11.3^Bd^	14.9^Bb^	14.2^Ba^	11.6^Bbc^	10.1^Bc^	13.4^Bab^
	EIP	17.3^Ba^	15.1^Bab^	13.1^Bbc^	11.9^Bc^	15.0^Ba^	13.1^Bab^	11.3^Bbc^	10.3^Bc^
SEM		5.07				5.21			
*P-value*	*A*	< 0.001				< 0.001			
	*D*	< 0.001				< 0.001			
	*A* × *D*	< 0.001				< 0.001			

*A*, effect of treatment; *D*, effect of silage days; *A* × *D*, interaction between treatment and silage days; Means with unlike lower case superscripts letters (a–c) differ among ensiling days (*P* < 0.05). Means with unlike upper case superscripts letters (*A*–*C*) differ among treatments (*P* < 0.05). SEM, standard error of mean. CP, CIP, EIP represent the silage of CK, CIL, EIL group respectively for 3, 7, 14, 28 days. NDS, natural detergent solute; ADL, acid detergent lignin; CL, cellulose; HC, hemicellulose; WSC, water-soluble carbohydrate

### Analysis of fermentation characteristics

**Fig. 1:**
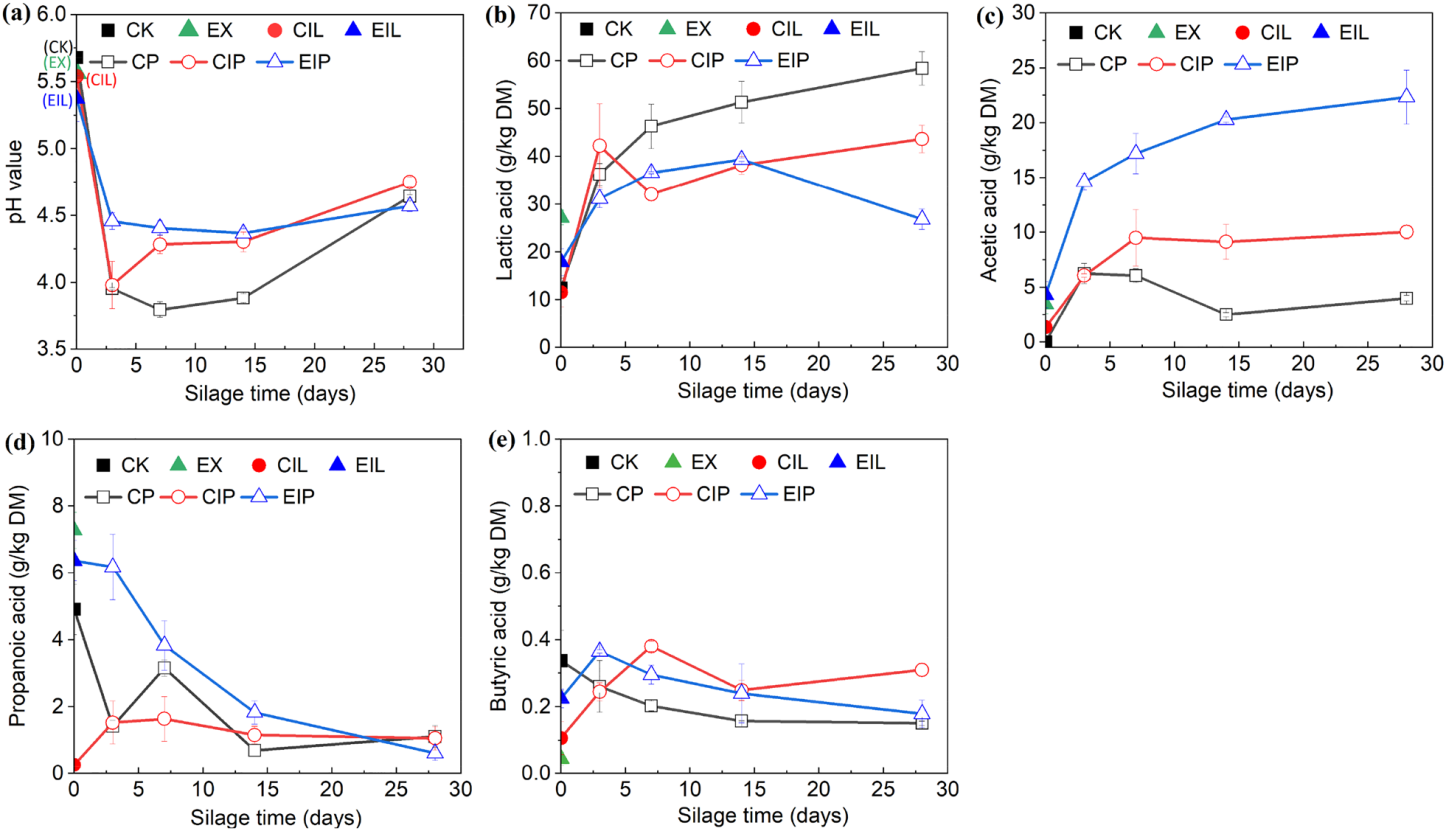
Dynamics of the pH value (**a**) and organic acids (**b–e**) in different treatments. CK, raw buckwheat straw; EX, expanded straw; CIL and EIL represent CK and EX groups treated with *I. lacteus* for 14 days respectively; CP, CIP, and EIP represent the silage of CK, CIL, EIL group respectively for 3, 7, 14, 28 days

The dynamic changes of pH and organic acids in different treatment processes are shown in Fig. 1, and the detailed analysis is shown in Additional file 1: Table S1. After expansion, the pH decreased from 5.68 in CK to 5.55 in EX. When the CK was treated with *I. lacteus* the pH showed a decrease from 5.68 to 5.54 (CIL), and the EX decreased from 5.55 to 5.37 (EIL). After 3 days of ensiling, the pH of all silage drastically decreased, with the CP and CIP silages decreasing to 3.95 and 3.98, and the EIP decreasing to 4.46. Subsequently, the pH in CP and CIP silages gradually rose to 4.75 and 4.64, respectively, on day 28, whereas the pH of the EIP silage remained relatively stable throughout the ensiling, reaching only 4.50 on day 28.

The dynamics of organic acids produced in different treatment processes are presented in Fig. 1b, c. The concentrations of lactic acid (LA), acetic acid (AA), and propionic acid (PA) in EX were significantly higher than those in CK, while butyric acid (BA) concentration was decreased. After fungal treatment, AA concentration increased to 1.50 g/kg DM in CIL and 4.33 g/kg DM in EIL, respectively. The EIL showed a decrease in LA and PA concentration compared to EX, and the PA in CIL almost disappeared, with the lowest LA content and no difference as compared to CK. After ensiling, the highest LA concentration was observed in CP silage, and ultimately 58.42 g/kg DM on day 28. The overall concentration of LA in EIP showed no significant difference when compared with CIP but decreased significantly after 14 days. The AA accumulated the fastest in EIP silage throughout the ensiling compared to others, leading to the highest AA concentration about 22.35 g/kg DM on day 28, followed by 10.05 g/kg DM in CIP silage, while the lowest concentration in CP was only 4.01 g/kg DM on day 28.

**Fig. 2:**
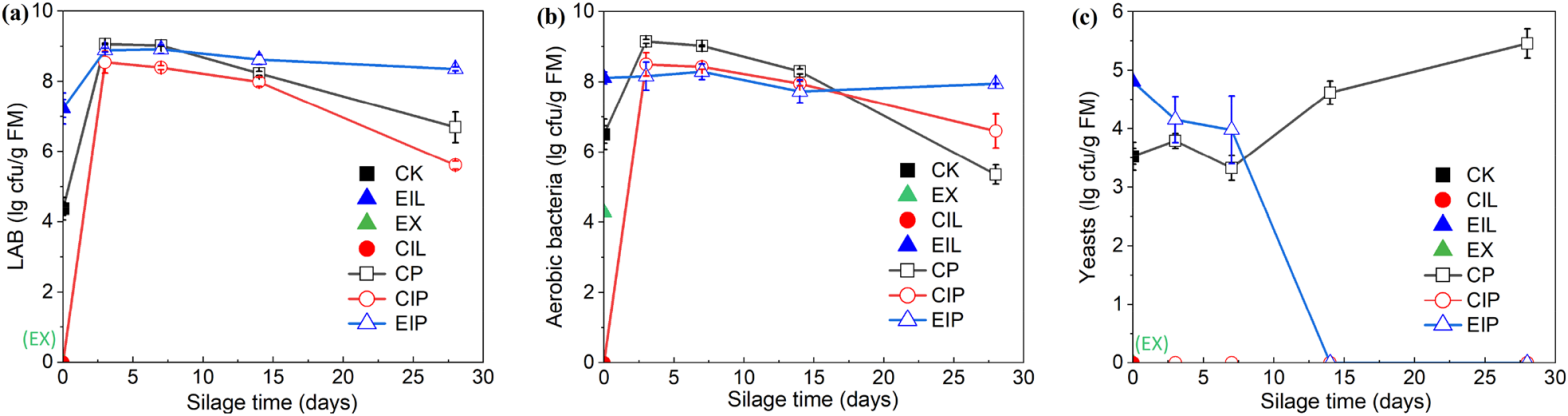
Microbial counts of LAB (**a**), aerobic bacteria (**b**), and yeasts (**c**) in different treatments. CK, raw buckwheat straw; EX, expanded straw; CIL and EIL represent CK and EX groups treated with *I. lacteus* for 14 days respectively; CP, CIP, and EIP represent the silage of CK, CIL, and EIL group respectively for 3, 7, 14, 28 days

The dynamic changes of microbial counts are shown in Fig. 2. The counts of LAB and yeasts of the samples in EX decreased below the detection limit (< 10^2^ cfu/FM), and the aerobic bacterial counts dropped from 6.50 in CK to 4.27 log_10_ cfu/g FM. After ensiling, the number of LAB and aerobic bacteria in CIP increased rapidly from 0 to about 8.50 log_10_ cfu/g FM and gradually decreased with the increase of silage days, and yeast was always below the detection limit. EIP silage had the highest number of LAB throughout the ensiling and did not decrease significantly in late silage as in CP and CIP silages. The yeast in EIP decreased rapidly after 7 days of silage and was below the detection line on day 14, while CP silage was still increasing after 7 days.

### Bacterial and fungal community

**Fig. 3:**
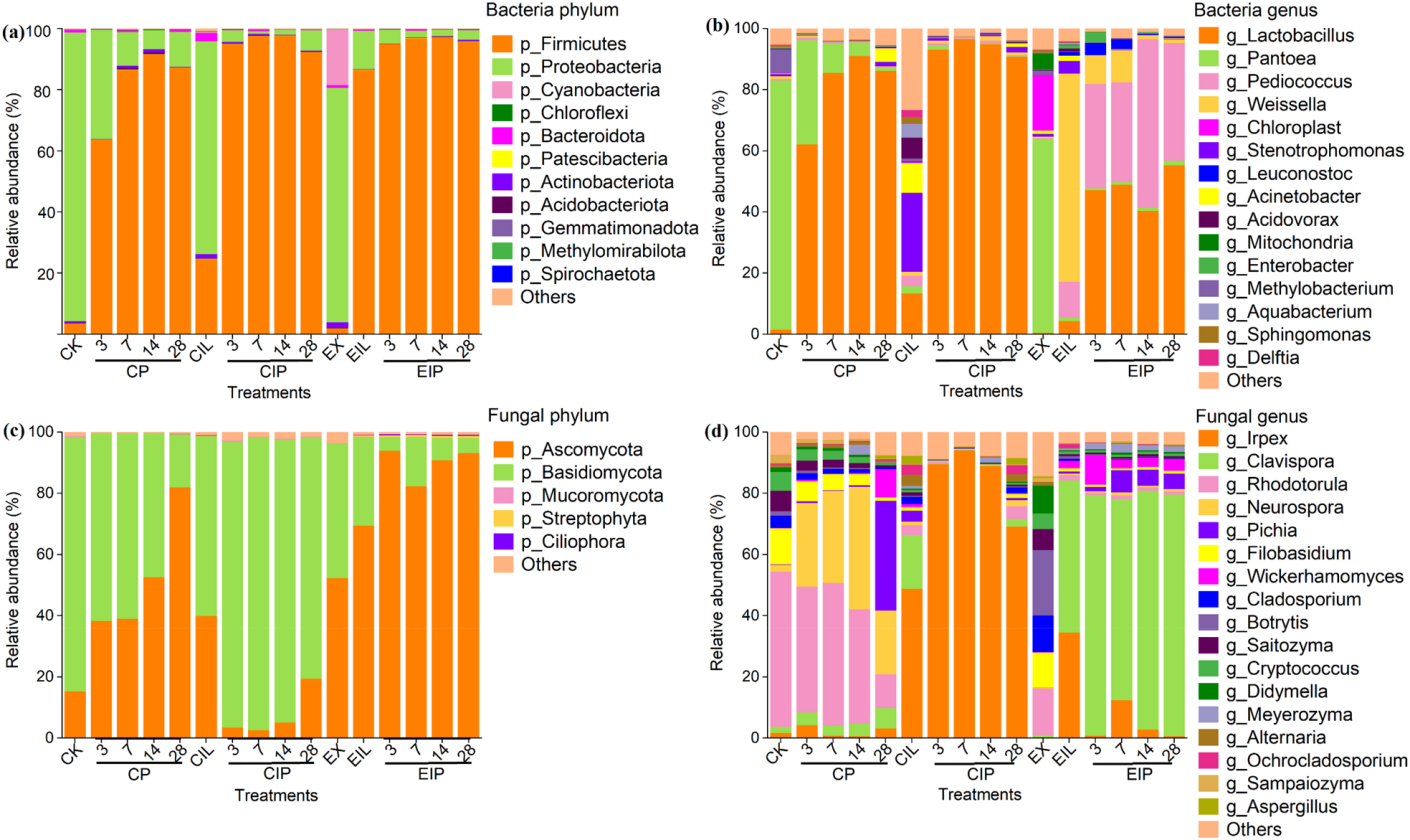
Relative abundance of bacterial (**a, c**) and fungal (**b, d**) communities at phylum and genus level. CK, raw buckwheat straw; EX, expanded straw; CIL and EIL represent CK and EX groups treated with *I. lacteus* for 14 days respectively; CP, CIP, and EIP represent the silage of CK, CIL, EIL group respectively for 3, 7, 14, 28 days

The relative abundance of bacterial and fungal communities at the phylum and genus level are shown in Fig. 3. For the bacteria, *Proteobacteria* was dominant in CK (94.50%) and EX (76.62%), and after fungal treatment, it was still dominant in CIL (69.53%), whereas *Firmicutes* was dominant phylum in EIL (86.54%) (Fig. 3a). During ensiling, *Firmicutes* could quickly dominate fermentation in CIP and EIP and accounted for 87.37% of CP, 92.33% of CIP, and 95.78% of EIP on day 28. At the genus level (Fig. 3b), *Pantoea* was the dominant genus in CK (81.48%) and was gradually replaced by *Lactobacillus* during silage. More colonies were detected in CIL, including *Stenotrophomonas* (25.85%), *Lactobacillus* (13.56%), *Acinetobacter* (9.62%), *Acidovorax* (6.69%), *Weissella* (1.19%) and *Pediococcus* (3.55%) et al. Unlike the CIL, the most dominant genus in EIL was *Weissella* (68.03%), followed by *Pediococcus* (11.56%) and *Lactobacillus* (4.37%). After ensiling, *Lactobacillus* could dominate the silage faster in CIP and occupied a larger proportion than CP silage, while EIP silage was co-dominated by *Pediococcus*, *Lactobacillus,* and *Weissella*.

For the fungi, the dominant phylum in CK was *Basidiomycota* (83.12%), and EX was dominated by *Ascomycota* (52.39%) (Fig. 3c). The dominant phylum of CIP and EIP was opposite, where *Basidiomycota* was the dominant phylum in CIP silage and *Ascomycota* was the highest in EIP silage. At the genus level (Fig. 3d), *Rhodotorula* (50.63%), *Filobasidium* (11.80%), and *Cryptococcus* (6.19%) were highly abundant in CK, and *Rhodotorula* decreased to 15.20% in EX. During terminal fungal treatment, the most abundant genus in the CIL was *Irpex* (48.90%), followed by *Clavispora* (17.57%) and *Pichia* (3.54%), but the abundance of *Clavispora* (49.62%) was higher than *Irpex* (34.66%) in EIL. In the CP silage, *Rhodotorula* increased with the extension of silage time, and *Pichia* was observed on day 28. *Irpex* and *Clavispora* were the dominant genera throughout the CIP and EIP silages, respectively.

### Bacterial functional prediction

**Fig. 4:**
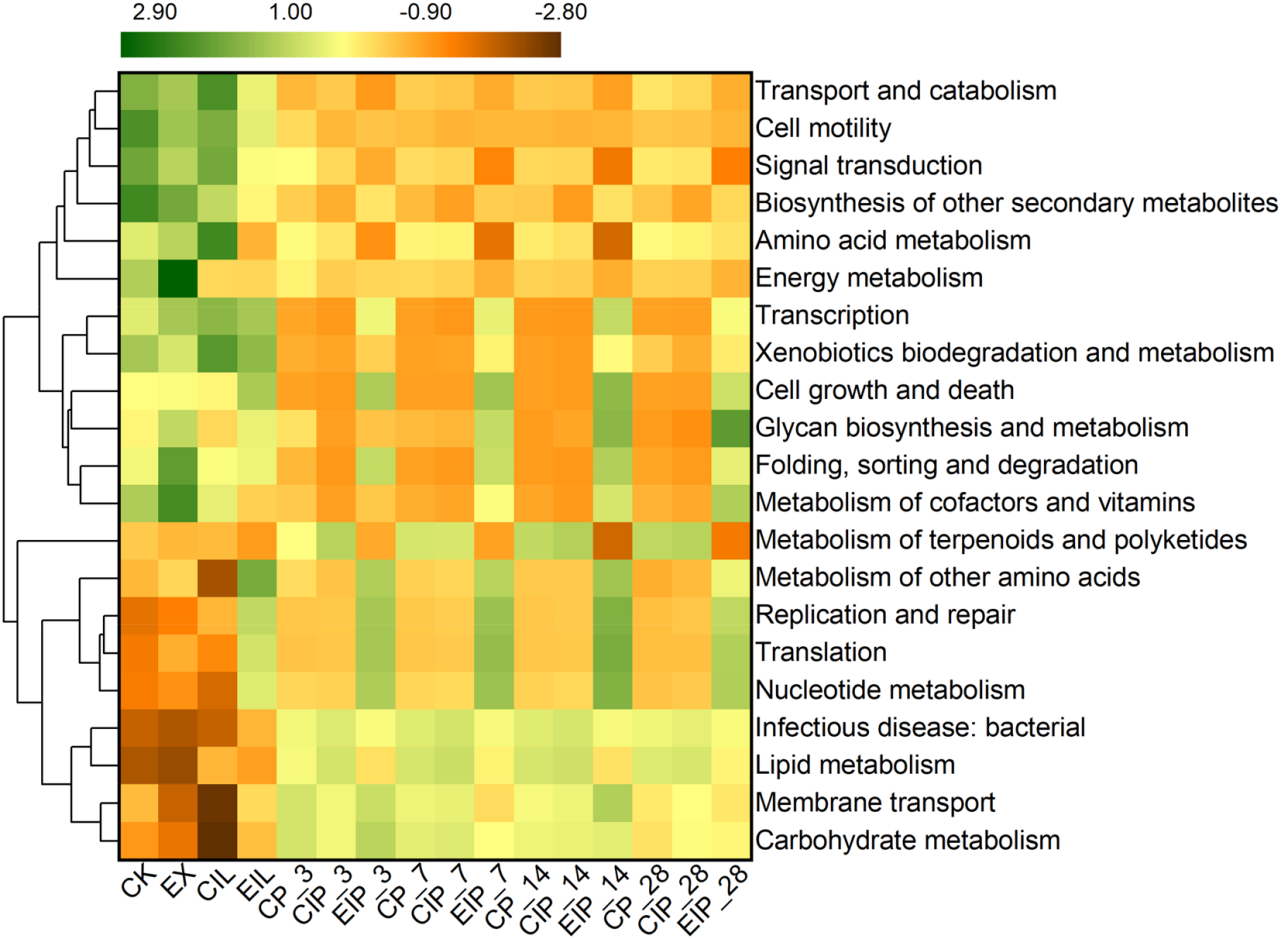
Heatmap of predicted functions of bacterial communities via PICRUSt. CK, raw buckwheat straw; EX, expanded straw; CIL and EIL represent CK and EX groups treated with *I. lacteus* for 14 days respectively; CP, CIP, and EIP represent the silage of CK, CIL, and EIL group respectively for 3, 7, 14, 28 days

According to the functional annotation, action path, and abundance information of samples in the database, the top 21 predicted functional abundance was drawn into the heatmap (Fig. 4). The EIL treatment increased carbohydrate and other amino acids metabolism, membrane transport, translation, and replication and repair, whereas the CIL treatment enriched the signal transduction, metabolism of terpenoids and polyketides, xenobiotics biodegradation, and amino acid. Ensiling increased carbohydrate and nucleotide metabolism, and decreased the relative abundance of cell motility, biosynthesis of other secondary metabolites, metabolism of xenobiotics biodegradation, cofactors and vitamins, energy, and amino acid. There was not much difference in metabolism between the CP and CIP silages. However, EIP silage downregulated the metabolism of membrane transport, amino acid, energy, lipid, carbohydrate, and terpenoids and polyketides, and upregulated genetic information processing, including translation, transcription, replication and repair, and nucleotide metabolism. Moreover, the metabolism of cofactors and vitamins and glycan biosynthesis were enriched in EIP and increased with the extension of silage days.

### Analysis of in vitro gas production

**Fig. 5:**
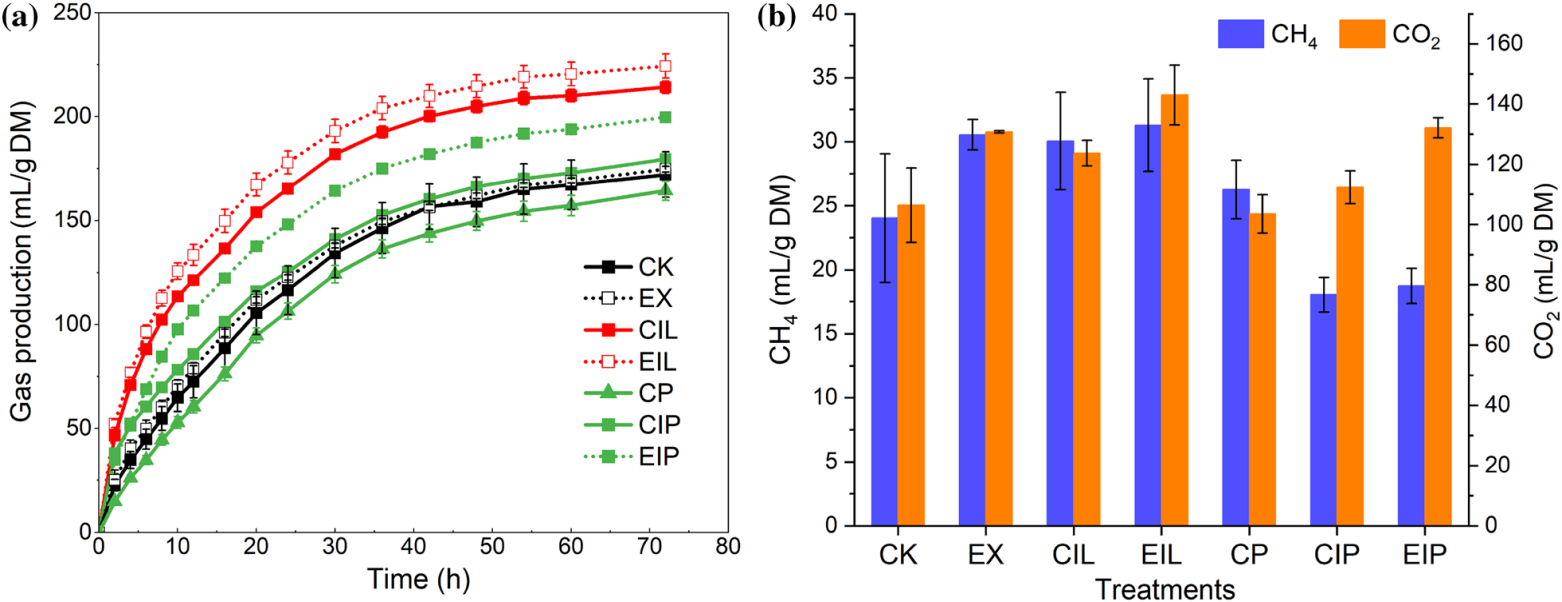
In vitro gas production (**a**) and the main gas component of CH_4_ and CO_2_ (**b**). CK, raw buckwheat straw; EX, expanded straw; CIL and EIL represent CK and EX groups treated with *I. lacteus*; CP, CIP, and EIP represent the silage of CK, CIL, and EIL group respectively for 28 days

Figure 5 revealed the total IVGP_72_, CH_4,_ and CO_2_ production of buckwheat straw in different treatment stages. Fungal degradation increased IVGP_72_, CH_4_, and CO_2_ production, with the highest level in expanded samples. Ensiling showed a decrease in IVGP_72_ but expanded straw still significantly retained the nutritional value of the straw compared with unexpanded samples. The CH_4_ production decreased significantly in CIP and EIP, and the replacement of expansion always had the highest level of CO_2_ production.

## Discussion

### Chemical composition analysis

Expansion could serve as a platform involving sterilization and disruption of structure for further biological pretreatment. By creating channels and opening the plant cell wall, expansion may provide opportunities for enzymes secreted by fungi, whose molecular weight is too large to enter an intact plant cell wall, to access and degrade lignin. The higher lignin degradation rate of expanded straw provided more NDS for silage fermentation. In CIP silage, the decrease of NDS was accompanied by the loss of CL and HC contents, which might be due to the activity of *I. lacteus* not being terminated, competing nutrition with LAB. In contrast, the activity of *I. lacteus* seemed to be well terminated in EIP silage to prevent nutrition loss. At the end of fungal fermentation in EIL, oxygen was gradually depleted and LAB began to proliferate and consume part of WSC. In contrast, due to the absence of microorganisms in the autoclaved sample of CIL, the initial level of ensiling in EIL was slightly lower. However, the WSC content of the CIP was consumed faster after ensiling and lower than that of the EIP silage. This was likely attributed to the higher microbial activity in CIP consuming more carbohydrates.

### Fermentation quality analysis

Fermentation parameters of different treatments could determine whether the combination of expansion and fungal treatment had better preservative effects. The shearing, extrusion, and the high temperature and pressure conditions destroyed the stability of lignocellulosic structure, weakened the intermolecular and intramolecular hydrogen bond, and dissociated more H^+^, resulting in a decrease in pH value of expanded samples. Chen et al. (2012) have reported that the partial hydrolysis of cellulose and hemicellulose in an acidic environment was conducive to the penetration of laccase into complex lignocellulose and enhanced the lignin degradation. Therefore, expansion provided more favorable conditions for lignin degradation by *I. lacteus* by damaging the complex structure of lignocellulose and decreasing the pH. At the initial stage of silage, due to the high residual WSC content in CIP and CP, it could be quickly utilized by LAB to decrease the initial pH of the environment. However, in the long run, the increase in NDS content in EIP was more meaningful for stabilizing pH during ensiling.

In this study, the increase of AA concentration and decrease of PA concentration in CIL and EIL suggested that *I. lacteus* potentially produced AA and degraded PA during fermentation. Some studies also reported acidification and organic acid production by white-rot fungi in a culture environment (Mao et al. 2021a, b; Mkel et al. 2002). The phenomenon of high levels of LA concentration in EX was unclear, which might be caused by a small number of high-temperature-resistant LAB fermenting easily obtained substrates after expansion when the extraction process formed an anaerobic environment. LA concentration subsequently decreased in EIL also might be related to fungal metabolism, as the result of fungal degradation of LA was also obtained in other research (Mao et al. 2021a, b). The PA concentrations of expanded samples were within the range of the high-quality silage (1–10 g/kg DM) (Agarussi et al. 2019). Expanded samples were more beneficial for inhibiting the growth of *Clostridium* and other harmful microorganisms during ensiling in terms of BA concentration. Untreated buckwheat straw inoculating with *L. plantarum*, which is a homogeneous fermented lactic acid bacteria, could rapidly ferment to produce LA (Mu et al. 2021). The decrease in pH value in CIP was consistent with the increase in LA content on day 3, indicating extensive lactic acid fermentation primarily occurred at the initial stage. Whereas in EIP silage, not only a high concentration of LA was produced, but also the highest suitable AA concentration, which was owing to different microbial communities with CP and CIP silage leading to different fermentation modes. The mixture of LA and AA seems to be better for fermentation and preservation of biomass, as the high level of AA concentration conferred antifungal activities against yeasts and molds (Li and Nishino 2013). Increased pH and lower AA concentration in CP and CIP silages on day 28 suggested weaker inhibition of undesirable microorganisms after fermentation.

The decrease of aerobic bacteria in EX and the disappearance of LAB, yeasts and molds reflected that the severe expansion condition had an optimistic sterilization effect or forced microbial dormancy. During fungal treatment, expanded straw provided more carbon and nitrogen sources to enhance the degradation of lignin by *I. lacteus*, which facilitated microorganism access to more easily fermentable substrates, thereby improving the growth environment of microorganisms and promoting the recovery of dormant spores. The almost constant number of LAB in EIP silage during anaerobic preservation suggested that the dominant LAB during ensiling were more acid-tolerant and more active, which was associated with a more stable pH. Although aerobic bacteria grew during EIL treatment, the populations remained almost unchanged after ensiling, unlike the large increase in other silages, which could reduce the competition for nutrients with LAB for colonization when the *Lactobacillus* was initially inoculated. CP silage kept higher yeast populations which might be due to the high LA concentrations during silage since yeasts readily utilize it for their propagation (dos Santos et al. 2018), and this was detrimental to the preservation of silage. This was in contrast to the fact that EIP silage could rapidly reduce yeast activity. Overall, the difference in initial conditions resulted in different fermentation modes of EIP and CIP during the subsequent silage process.

### Bacterial and fungal community analysis

The differences in fermentation parameters and nutrients in different treatments were related to microorganisms. On the phylum level, *Proteobacteria* are usually the dominant phyla before silage (Xian et al. 2022), such as in CK, EX, and CIL groups. Interestingly, *Cyanophyta* was the subdominant phylum in EX. *Cyanobacteria* are the only oxygenic phototrophic bacteria, and an important property is the ability to fix atmospheric N_2_ (Singh et al. 2016). It can produce a unique nitrogenous compound known as cyanophycin or multi-L-arginyl-poly (L-aspartic acid), and its high nitrogen content means that it can serve as a nitrogen reservoir (Mackerras et al. 1990). This finding provided an advantage to the nitrogen source requirement for *I. lacteus* growth. Most LAB belong to *Firmicutes* and expanded straw promoted the degradation of lignocellulose by *I. lacteus*, providing more nutrients for the proliferation of LAB and reducing the pH. The decrease of pH before ensiling was beneficial to ensilage. In conclusion, the combination of expansion and *I. lacteus* promotes the growth of *Firmicutes* and inhibits the growth of *Proteobacteria*.

To further study the different treated processes on the microorganism community, we examined the microbial community at the genus level. *Pantoea* and *Methylobacterium* are considered undesirable in silage and increase the fermentation and quality losses of silage (Avila and Carvalho 2020). *Pantoea* is a genus separated from *Enterobacter* and has parthenogenic anaerobic properties, which can compete with LAB for nutrients during silage, resulting in nutrient losses to cause butyric acid accumulation (Bai et al. 2023; Li et al. 2017). *Methylobacterium* are aerobic, Gram-negative, and facultatively methylotrophic bacteria that survive in variable environments (Kato et al. 2008). *Methylobacterium* disappeared due to the low pH value of CP silage, but *Pantoea* still survived in the early stage of silage. Expansion could effectively decrease or inhibit both genera. Since the lignocellulosic degrading enzyme secreted by* I. lacteus* catalyzed lignin oxidation while consuming molecular oxygen, and the high temperature and pressure during expansion also extracted a large amount of fermentable sugars (Souza et al. 2022), low oxygen conditions were easily formed in the later stage of EIL treatment, thereby promoting the growth of LAB. *Weissella* and *Pediococcus* are known to function as the early initiators of lactic acid fermentation (Bao et al. 2022; You et al. 2021). *Weissella* is an obligately heterofermentative anaerobic bacterium with the ability to convert WSC to AA and LA during ensiling (Xian et al. 2022). Whereas in CIL, *Stenotrophomonas* and *Acinetobacter* are both non-fermentative aerobic bacteria with low nutrient requirements (Kämpfer and Glaeser 2012), which also reflected the less nutrition from fiber degradation by *I. lacteus* on unexpanded substrate. After ensiling, the relative abundance of *Weissella* was significantly decreased in EIP silage owing to its low acid tolerance and even disappeared on day 14. *Pediococcus*, due to the founder effect in EIL and its advantage of growing faster than *Lactobacillus* (Alhaag et al. 2019; Porto et al. 2017), could co-dominate silage with *Lactobacillus*, and it has been proven to promote lactic acid fermentation and improve the preservation of nutrients (Blajman et al. 2020).

For the fungi, inoculation with *I. lacteus* could inhibit the growth of *Neurospora* in silage. Expansion could significantly decrease the relative abundance of *Rhodotorula*, indicating its weak resistance to harsh environments. At the end of fungal treatment, the relative abundance of *Irpex* in expanded straw was about 14% lower than that in autoclaved straw, but a higher lignin degradation rate occurred in EIL. Therefore, we cannot rule out that *Irpex* was the dominant bacterial genus within 14 days and was gradually replaced by *Clavispora* with the rapid degradation of lignin. *Clavispora* is a non-conventional yeast and exhibits native tolerance to some fermentation inhibitors commonly observed in lignocellulosic hydrolysates (Liu et al. 2012). This new yeast produces sufficient native *β*-glucosidase enzyme activity allowing it to grow on cellobiose as a sole source of carbon (Liu et al. 2012). Our previous studies reported that the *β*-glucosidase activity secreted by *I. lacteus* in the early stage of the combined treatment of expansion and *I. lacteus* is low, while the exoglucanases activity is high, which can cut the cellobiose units from the reducing end or non-reducing end of the polysaccharide chain (Reese et al. 1950). Hence, the more retained cellobiose possibly stimulated the growth of *Clavispora*. *β*-glucosidase can digest cellobiose into simple sugar glucose for other microorganisms to utilize, which may be the reason for the colonization of *Weissella* and *Pediococcus* in EIL. Ensilage further promoted the proliferation of *Clavispora* in EIP groups, which might be caused by the founder effect in EIL. In contrast, the increased abundance of *Irpex* in CIP silage was possibly from irritation of substrate, when more degradation of surface hemicellulose and cellulose in CIL led to increased lignin exposure. However, the lignin degradation did not increase along with the abundance of *I. lacteus* during ensiling, because the lignin-degrading enzymes required the consumption of molecular oxygen (Bao et al. 2023) and the optimal pH value of laccase was about 4.5–5. However, the significant decrease in HC and CL contents in CIP silage might be owing to the activity of *I. lacteus*. Both *Irpex* and *Clavispora* showed good acid resistance throughout the ensiling.

### Potential function of microbial community

The heatmap of predicted functions implied that the samples related to expansion significantly affected the major functions of the bacterial community. The increased amino acid metabolism and energy metabolism of expanded straw might be due to the presence of *Cyanophyta*, which have high nitrogen metabolism and nitrogen fixation capacity. Expanded samples degraded by *I. lacteus* showed higher levels of carbohydrate metabolism. Carbohydrate metabolism mainly contained gluconeogenesis and glycolysis metabolism, thus it was inferred that on the one hand, expanded straw caused a higher ability of lignocellulose degradation by *I. lacteus* to produce carbohydrates. On the other hand, more bacteria genera in EIL that use WSC, such as *Weissella*. Bai et al. (2021) considered that the relative abundance of total LAB in microbial communities affects the abundance of carbohydrate metabolic pathways. According to the report of Kilstrup et al. (2005), most metabolic reactions are related to either bacterial utilization of nucleotides or their regulation by metabolites. Silage fermentation is dominated by LAB, therefore, the enhancement of carbohydrate and nucleotide metabolism might be related to the increased abundance of LAB. The highest nucleotide metabolism in expanded straw silage might be attributed to gene levels in the high-abundance *Pediococcus* and *Weissella*. Although the dominant *Lactobacillus* in CIP and CP silages led to the highest amino acid metabolism (Okoye et al. 2022), which was consistent with high concentrations of LA in CP and CIP since the formation of LA involves the decarboxylation of amino acids and the deamination of arginine (Bai et al. 2021). In addition, expanded buckwheat straw might contribute to increasing vitamin yield and glycan biosynthesis during ensiling.

### In vitro* gas production analysis*

IVGP highly depends on the degradability of soluble components in the incubated substrates and the partitioning of fermented substrates to microbial biomass production (Elghandour et al. 2017). Besides, non-soluble carbohydrate fraction and the biomass structure also affect gas production. Expansion promoting the release of soluble components from the cell wall matrix to increase NDS content and enhancing lignin degradation by *I. lacteus* was the most plausible explanation for the higher IVGP of expanded samples. The fermented substrate associated with lignin limited the utilization of feedstock, resulting in low conversion efficiency when directly ensiled. CH_4_ and CO_2_ are the main products in livestock industry, comprising approximately 14.5% of global anthropogenic greenhouse gas emissions, with CH_4_ having a global warming potential 28-fold greater than the impact of CO_2_ (Monteiro et al. 2022). Ensiling after fungal treatment shifted fermentation toward less CH_4_ production possibly due to the lower pH inhibiting the methanogenic activity (Zhang et al. 2020), which is a potential strategy to reduce greenhouse gas emissions in livestock production. Although ensiling caused a certain nutrition loss in terms of IVGP_72_, expansion seemed to make up for this deficiency, suggesting that expansion pretreatment was more conducive to improving the utilization value and preservation of straw waste.

### Evaluation of the composite processing technology

The composite treatment technology conducted in this study runs through the pretreatment and preservation of biomass. The pretreatment method inevitably accompanies energy consumption, and the largest energy consumption of this technology is the operation of the expanding machine, with a power of approximately 36 kW. However, the energy requirement of expansion is much lower than that of similar treatment methods such as steam explosion and ammonia fiber explosion. Furthermore, continuous operation and no additional processing save more economic costs. Silage and fungal treatments hardly involve energy consumption and are the lowest cost and environmentally friendly treatments. However, there are also some drawbacks of expansion sterilization. The short expansion time led to the revival of some high-temperature resistant spores, which could consume partial WSC in subsequent fungal treatment. Therefore, in the subsequent research, different expansion conditions can be set to further optimize the sterilization effect for fungal treatment.

## Conclusion

The severe expansion conditions created an opportunity for colonization of *I. lacteus* and enhanced lignin degradation. The increased lignin degradation of expanded straw increased the soluble components for ensiling. Different initial conditions caused by fungal treatment for expanded and autoclaved straw led to different fermentation patterns and microbial communities during ensiling. EIP silage was dominated by *Lactobacillus*, *Pediococcus*, and *Weissella* and had high LA and AA concentrations and stable pH, while unexpanded straw was only dominated by *Lactobacillus*. *Clavispora* gradually replaced *Irpex* in EIP, which potentially promoted LAB growth and AA production. This innovative way of recycling straw can achieve more nutrient retention when the straw is stored and reduce CH_4_ emissions. The advantages of continuity and ease of scale-up of expansion enhance the practicality and economy of the technology.

### Supplementary Information

**Additional file 1: Table S1.** Dynamics of the pH value and organic acids throughout the process.

## Data Availability

Data are available on request to the authors.
