# Supplementary material for: Enhanced lignin degradation by Irpex lacteus through expanded sterilization further improved the fermentation quality and microbial community during the silage preservation process
Source: Bioresour Bioprocess. 2024 Jan 22;11(1):14. doi: 10.1186/s40643-024-00730-2 (PMC10992542; doi:10.1186/s40643-024-00730-2)
Supplement: Supplementary file 1 — Additional file 1: Table S1. Dynamics of the pH value and organic acids throughout the process. [file 40643_2024_730_MOESM1_ESM.docx]

**Table S1.** Dynamics of the pH value and organic acids throughout the process.

| Item | Groups^1^ | Silage days (d)^2^ | | | | | SEM | *P-value* | | |
| --- | --- | --- | --- | --- | --- | --- | --- | --- | --- | --- |
|  |  | 0 | 3 | 7 | 14 | 28 |  | A | D | A×D |
| pH | CK | 5.68^Aa^ | 3.95^Bb^ | 3.79^Bc^ | 3.88^Bbc^ | 4.64^ABa^ | 0.892 | <0.001 | <0.001 | <0.001 |
|  | EX | 5.56^A^ | -- | -- | -- | -- |  |  |  |  |
|  | CIL | 5.54^Aa^ | 3.98^Bc^ | 4.28^Ac^ | 4.31^Ac^ | 4.75^Ab^ |  |  |  |  |
|  | EIL | 5.37^Ba^ | 4.45^Ab^ | 4.40^Ab^ | 4.36^Ab^ | 4.57^Bb^ |  |  |  |  |
| LA | CK | 12.43^Bc^ | 36.28^ABb^ | 46.32^Aab^ | 51.34^Aab^ | 58.46^Aa^ | 1.930 | <0.001 | <0.001 | <0.001 |
|  | EX | 27.00^A^ | -- | -- | -- | -- |  |  |  |  |
|  | CIL | 11.55^Bb^ | 42.24^Aa^ | 32.08^Ba^ | 38.20^Ba^ | 43.68^Ba^ |  |  |  |  |
|  | EIL | 17.94^ABc^ | 31.18^Bab^ | 36.63^ABa^ | 39.37^Ba^ | 26.83^Cb^ |  |  |  |  |
| AA | CK | 0.00^Bc^ | 6.25^Ba^ | 6.07^Ba^ | 2.50^Cb^ | 4.01^Bab^ | 0.987 | <0.001 | <0.001 | <0.001 |
|  | EX | 3.48^A^ | -- | -- | -- | -- |  |  |  |  |
|  | CIL | 1.33^ABb^ | 6.07^Bab^ | 9.51^ABa^ | 9.14^Ba^ | 10.04^Ba^ |  |  |  |  |
|  | EIL | 4.32^Ac^ | 14.63^Ab^ | 17.20^Aab^ | 20.29^Aab^ | 22.35^Aa^ |  |  |  |  |
| PA | CK | 4.91^A^ | 1.42^B^ | 3.16^AB^ | 0.70^B^ | 1.11^A^ | 0.337 | <0.001 | <0.001 | <0.001 |
|  | EX | 7.27^A^ | -- | -- | -- | -- |  |  |  |  |
|  | CIL | 0.26^Bb^ | 1.53^Ba^ | 1.64^Ba^ | 1.16^ABab^ | 1.06^Aab^ |  |  |  |  |
|  | EIL | 6.36^Aa^ | 6.17^Aa^ | 3.83^Aab^ | 1.83^Abc^ | 0.61^Bc^ |  |  |  |  |
| BA | CK | 0.33^Aa^ | 0.26^Bab^ | 0.20^Cbc^ | 0.16^c^ | 0.15^Bc^ | 0.014 | <0.001 | <0.001 | <0.001 |
|  | EX | 0.05^C^ | -- | -- | -- | -- |  |  |  |  |
|  | CIL | 0.11^BCb^ | 0.24^Bab^ | 0.38^Aa^ | 0.25^ab^ | 0.31^Aa^ |  |  |  |  |
|  | EIL | 0.22^ABb^ | 0.36^Aa^ | 0.29^Bab^ | 0.23^b^ | 0.17^Bb^ |  |  |  |  |

A, effect of treatment; D, effect of silage days; A × D, interaction between treatment and silage days; Means with unlike lower case superscripts letters (a-c) differ among ensiling days (*P* < 0.05). Means with unlike upper case superscripts letters (A-C) differ among treatments (*P* < 0.05). SEM, standard error of mean. ^1^Groups: CK, feedstock; EX, expanded straw; CIL and EIL represent CK and EX groups treated with *I. lacteus* for 14 days respectively. ^2^Silage days: CK, EX, CIL, and EIL group ensiled with *L. plantarum* for 0, 3, 7, 14, and 28 days. LA, lactic acid; AA, aerobic acid; PA, propionic acid; BA, butyric acid;
